# Ixekizumab: an alternative for HIV-positive psoriasis patients

**DOI:** 10.1186/s12981-024-00675-8

**Published:** 2024-11-28

**Authors:** Fanghua Liu, Zhou Liu, Rongming Yang, Dandan Huang, Yongzhi Han

**Affiliations:** 1 Department of Dermatology, Ganzhou Municipal Hospital, Ganzhou, Jiangxi Province 341000 People’s Republic of China; 2 Department of Dermatology, Guangdong Provincial People’s Hospital, Guangdong Academy of Medical Sciences, No.106, Zhongshan Second Road, Guangzhou, Guangdong Province 510080 People’s Republic of China

**Keywords:** Psoriasis, HIV, Ixekizumab

## Abstract

Psoriasis is a recurrent and protracted inflammatory disease. Generalized erythema, plaques, and silvery scales on the surface mainly characterize its skin lesions. Biologics bring new hope to psoriasis patients. However, HIV infection is a major concern before receiving biologics. Here, we present a case of the efficacy and safety of Ixekizumab, an interleukin 17 inhibitor, in the treatment of psoriasis patients living with HIV (PPLHIV).

**Supplementary Information:**

The online version contains supplementary material available at 10.1186/s12981-024-00675-8.

## Introduction

### Case presentation

**Fig. 1:**
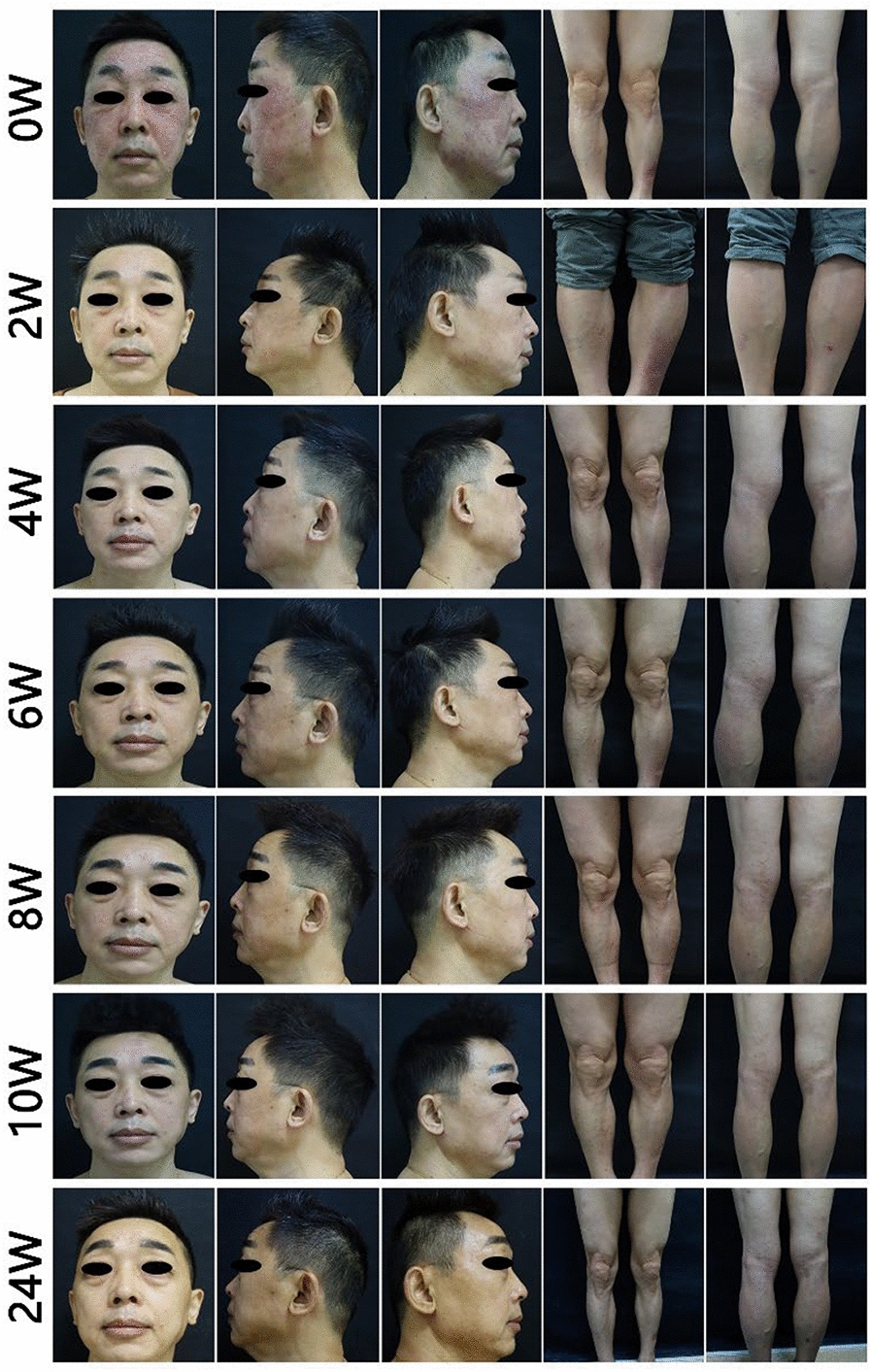
Ixekizumab treatment photographs. Ixekizumab treatment started (erythema and thin scales distributed on the face and lower legs), the second week (facial erythema and scales significantly subsided), the fourth week (complete clearance of facial lesions, fading color of leg erythema, and reduction of scales), the sixth week (complete clearance of lesions), the eighth week (no new onset of facial rashes, small patchy erythematous patches on the lower legs), and the tenth week (complete clearance of rashes, and lasts until week 24 with no new onset were found)

**Fig. 2:**
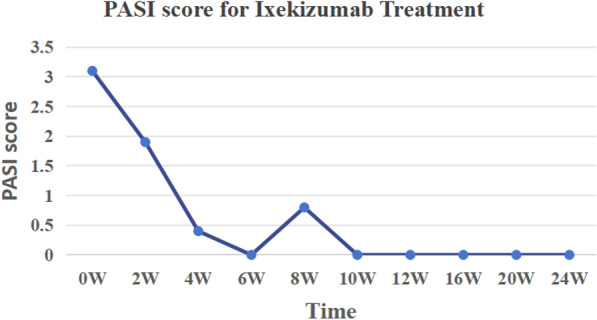
PASI Score for Ixekizumab Treatment

The patient was a 51-year-old man. HIV-1 was found to be positive in 2014, and western blot results identified p17, p24, p31, p51, p55, p66, gp41, gp120, and gp160. Highly active antiretroviral therapy (HAART) with undetectable viral load was immediately started. In 2021, the patient developed erythema and scaling on the face and lower limbs, recurrent rash, and no involvement of nails or joints. He had been diagnosed with psoriasis at another hospital, and the rash subsided after 1 month of oral cyclosporine treatment. Due to concerns about cyclosporine-induced nephrotoxicity, hypertension, and especially suppression of T-cell immunity, cyclosporine was discontinued one month later, and the patient's condition recurred. In November 2023, he came to the clinic, and the patient's skin lesions were mainly distributed on the face (Fig. 1), which seriously affected the patient's social life, self-confidence, and quality of life. Baseline hematology and metabolic laboratory results were normal. HBsAg, HBsAb, HBeAg, HCV-Ab, and T-SPOT were negative, HBeAb and HBcAb were positive, and HBV DNA was < 100.00 IU/mL. The Counts of CD4 T lymphocyte and CD8 T lymphocyte were 641 cells/ μl and 654 cells/μl, respectively, the CD4/CD8 ratio was 0.8, and HIV viral load was not detected. No infections were found in the lungs, digestive tract, and other areas. In the context of anti-HIV therapy, 160 mg of Ixekizumab was administered subcutaneously at week 0, followed by 80 mg at weeks 2, 4, 6, 8, 10, and 12, and then maintained at 80 mg every 4 weeks. The patient's skin lesions were completely cleared at week 10 and currently maintained on Ixekizumab for seven months with no episodes of skin lesions observed (Fig. 2). Laboratory data in June 2024 showed a CD4 T lymphocyte count of 618 cells/μL, a CD8 T lymphocyte count of 609 cells/μL, the CD4/CD8 ratio was 1.02, and HIV viral load was not detected (Table 1). No infection was found.

**Table 1:** HIV parameters (CD4, CD8, and viral load) before and during Ixekizumab

Date(mmm/yyyy)	May 2023	Feb 2024	Jun 2024
HIV viral load(copies/ml)	TND	TND	TND
CD4(cells/μl)	528	641	618
CD8(cells/μl)	675	654	609
CD4/CD8 Ratio	0.78	0.98	1.02

TND status, target not detected (HIV viral load < 20 copies per ml)

## Discussion

Psoriasis is a common skin disease in HIV-infected individuals, with guttate, reverse, and erythrodermic psoriasis being more common. It can appear at any stage of HIV infection and varies in severity from mild to severe [1]. In the context of HIV infection, psoriasis patients present with atypical clinical manifestations, more severe disease, and more recalcitrant treatment as CD4 + T cell counts decrease [2]. PPLHIV are at risk of opportunistic infections in T-cell-depleted settings. HIV is therefore considered a contraindication to immunosuppressive therapy in psoriasis [3]. so drugs such as cyclosporine and methotrexate were not the preferred treatment options for this patient. According to guidelines, biological agents can be used with caution when HIV is adequately treated and monitored [4]. Treatment of PPLHIV with biologic therapy does not significantly affect HIV viral load, CD4 cell count, CD4 proportion, and infection rate during the first 12 months of treatment [5]. TNF-α is overexpressed at all stages of HIV infection, increasing viral load and disease progression with TNF-α elevation [6]. Data suggests that TNF-α is a safe and effective treatment option for HIV-infected individuals, and the selection of TNF-α antagonists should ensure CD4 T lymphocyte counts exceeding 200/μL [7]. 2 HIV patients with erythrodermic psoriasis received anti-IL-17 therapy and obtained complete lesion clearance, with no recurrent or opportunistic infections during treatment [8]. Another HIV patient with articular psoriasis was treated with anti-IL-17, which significantly improved skin lesions and joint symptoms, along with an increased CD4 T cell count and an undetectable HIV-1 viral load [9]. Guidelines from the American Academy of Dermatology and National Psoriasis Foundation (AAD-NPF) recommend that anti-il-17 monoclonal antibodies can be selected for HIV patients who are receiving antiretroviral therapy and have a controlled viral load [10, 11]. In the pathophysiology of psoriasis, IL-17 is a "peripheral" cytokine. Anti-IL17 seems to be comparatively safe in comparison to other biologics. To date, there have been few cases of HIV-associated psoriasis treated with Ixekizumab. In this case, the patient had good immune function, no viral load, and the efficacy rapidly and significantly improved the quality of life after treatment. During the seven-month follow-up, there was no impact on the HIV viral load and T cell count, and no infection occurred. Although TNF-α, IL-17, and IL-23 inhibitors have fewer opportunistic infections in non-HIV patients^12^, meta-analyses have ostensibly shown some rates of reactivation in HIV using biologic therapies [13]. PPLHIV should consult an infection specialist and closely monitor for potential adverse events while being treated.

## Supplementary Information

Additional file 1.

## Data Availability

No datasets were generated or analysed during the current study.

## References

[1] Alpalhão M, Borges-Costa J, Filipe P. Psoriasis in HIV infection: an update. Int J STD AIDS. 2019;30(6):596–604. 30813860 10.1177/0956462419827673

[2] Ceccarelli M, et al. HIV-associated psoriasis: epidemiology, pathogenesis, and management. Dermatol Ther. 2019;32(2): e12806. 30588732 10.1111/dth.12806

[3] Kaushik SB, Lebwohl MG. Psoriasis: which therapy for which patient: Focus on special populations and chronic infections. J Am Acad Dermatol. 2019;80(1):43–53. 30017706 10.1016/j.jaad.2018.06.056

[4] Smith CH, et al. British Association of Dermatologists guidelines for biologic therapy for psoriasis 2017. Br J Dermatol. 2017;177(3):628–36. 28513835 10.1111/bjd.15665

[5] Xu, J., et al., *The impact of psoriasis biologic therapy on HIV viral load and CD4(+) cell counts in HIV-positive individuals: A real-world cohort study.* J Eur Acad Dermatol Venereol, 2023. 10.1111/jdv.19020 36897246

[6] Morar N, et al. HIV-associated psoriasis: pathogenesis, clinical features, and management. Lancet Infect Dis. 2010;10(7):470–8. 20610329 10.1016/S1473-3099(10)70101-8

[7] Myers B, et al. Biologic treatment of 4 HIV-positive patients: a case series and literature review. J Psoriasis Psoriatic Arthritis. 2021;6(1):19–26. 35784673 10.1177/2475530320954279 PMC9249044

[8] Pangilinan MCG, Sermswan P, Asawanonda P. Use of anti-IL-17 monoclonal antibodies in HIV patients with erythrodermic psoriasis. Case Rep Dermatol. 2020;12(2):132–7. 32999648 10.1159/000508781 PMC7506271

[9] Qian F, et al. Use of ixekizumab in an HIV-positive patient with psoriatic arthritis. Int J STD AIDS. 2022;33(5):519–21. 35220812 10.1177/09564624221076289

[10] Menter A, et al. Joint AAD-NPF guidelines of care for the management and treatment of psoriasis with biologics. J Am Acad Dermatol. 2019;80(4):1029–72. 30772098 10.1016/j.jaad.2018.11.057

[11] Sood, S., et al., *Use of biologic treatment in psoriasis patients with HIV: A systematic review.* J Am Acad Dermatol, 2024. 10.1016/j.jaad.2024.02.042 38452816

[12] Penso L, et al. Association between biologics use and risk of serious infection in patients with psoriasis. JAMA Dermatol. 2021;157(9):1056–65. 34287624 10.1001/jamadermatol.2021.2599 PMC8295892

[13] Li L, et al. Reactivation rates of hepatitis B or C or HIV in patients with psoriasis using biological therapies: a systematic review and meta-analysis. Clin Exp Med. 2023;23(3):701–15. 35499793 10.1007/s10238-022-00827-y

