# Supplementary material for: Ixekizumab: an alternative for HIV-positive psoriasis patients
Source: AIDS Res Ther. 2024 Nov 28;21:87. doi: 10.1186/s12981-024-00675-8 (PMC11603802; doi:10.1186/s12981-024-00675-8)
Supplement: Supplementary file 1 — Additional file 1. [file 12981_2024_675_MOESM1_ESM.pdf]

患者对其资料发表于国内外学术期刊的知情同意书

本人何大明同意我的资料出现在学术刊物和相关出版物。我已看过文中所用图片并且了解了发表的内容。

我已获知：

1、我的名字不会被公开。但我也理解不能保证完全保密。

2、我的资料会发表于全球性的网站和期刊上，印刷版本和网络版本会供医生、媒体、大众阅读。

3、我的资料不会被用于商业用途。

4、我知道这些影像资料会用于向医学界或公众宣传疾病的特征性表现、治疗方法及效果、病程进展、诊治难点和其他相关问题。

Patient's consent for the publication of material relating to him/she in domestic and international academic journals

I give my consent for this material to appear in academic journals and associated publications. I have seen any pictures and read the material to be published.

I understand that:

1.My name will not be published. I understand, however, that complete anonymity cannot be guaranteed.

2.The material may be published and placed on worldwide website and journals. Both the printed version and the website are seen and read by doctors, journalists, and members of the public.

3.The material will not be used for advertising or packaging.

4.I understand that such imaging records may be used for the purpose of informing the medical profession or the general public about characteristics of this disease and its diagnosis, prognosis, treatment and other related issues.

患者/监护人（Patient/Guardian） He da Ming

日期（date） 22 - May - 2024

医生（physician） Han yong zhi
